# Preparation and Characterization of Modified ZrO_2_/SiO_2_/Silicone-Modified Acrylic Emulsion Superhydrophobic Coating

**DOI:** 10.3390/ma16247621

**Published:** 2023-12-13

**Authors:** Jiaxin Ben, Peipei Wu, Yancheng Wang, Jie Liu, Yali Luo

**Affiliations:** 1 College of 2011, Nanjing Tech University, Nanjing 211800, China; 2 College of Materials Science and Engineering, Nanjing Tech University, Nanjing 211800, China

**Keywords:** super hydrophobic coating, spray-coating method, silica, modified zirconium dioxide, silicone-modified acrylic emulsion

## Abstract

Superhydrophobic coatings have increasingly become the focal point of research due to their distinctive properties like water resistance, wear resistance, and acid-base resilience. In pursuit of maximizing their efficiency, research has primarily revolved around refining the fabrication process and the composition of emulsion/nanoparticle coatings. We innovatively devised a superhydrophobic coating by employing a spraying technique. This involved integrating a γ-Methacryloyloxypropyltrimethoxysilane (KH570)-modified ZrO_2_/SiO_2_/silicone-modified acrylic emulsion. A comprehensive evaluation of this coating was undertaken using analytical instruments such as Fourier transform infrared spectroscopy (FTIR), X-ray diffraction (XRD), scanning electron microscopy (SEM), energy dispersive spectroscopy (EDS), and confocal laser scanning microscopy (CLSM). The coating demonstrated exceptional performance across a range of tests, including wear, immersion, and anti-icing cleaning, showcasing notable wear resistance, sodium chloride corrosion resistance, self-cleaning efficiency, and thermal stability. In particular, one coating exhibited super-hydrophobic properties, with a high contact angle of 158.5 degrees and an impressively low rolling angle of 1.85 degrees. This remarkable combination of properties is attributed to the judicious selection of components, which significantly reinforced the mechanical strength of the coating. These enhancements make it highly suitable for industrial applications where self-cleaning, anti-icing, and anti-contamination capabilities are critical.

## 1. Introduction

Superhydrophobic coatings are notable for their ability to achieve contact angles greater than 150° for droplets, while simultaneously maintaining a rolling angle below 5° [1]. Such characteristics render them invaluable in various applications, including oil-water separation [2,3], anti-icing mechanisms [4,5], chemical analysis [6,7], antimicrobial medical solutions [8], and solar panels [9]. These coatings are engineered by combining emulsion surfaces with nanoparticle overlays, ensuring both optimal surface roughness for air entrapment and minimal surface activation energy to repel water molecules.

Different methodologies, such as etching [10,11,12], templating [13,14,15], sol-gel [16,17,18], spray coating [19,20,21], spin coating [22,23,24], and electrospinning [25,26,27], have been investigated to develop superhydrophobic coatings. Among the materials employed, nanoparticles are favored for their inherent hardness and distinct micro-nanostructures, which have proven pivotal in generating superhydrophobic surfaces. In terms of industrial production, it is imperative that these coatings exhibit both robust adhesion to substrates and remarkable mechanical resilience. To ensure these features, silicone-modified acrylic emulsion are utilized as binders, effectively binding the nanoparticles to the substrate. This structure is known as the “substrate/binder/superhydrophobic nanoparticle” system.

However, there remain challenges to address, especially when considering applications like medical implants, seawater desalination, and cable transmission. These applications demand coatings that resist acids and bases, exhibit anti-icing efficiency, and maintain self-cleaning attributes. To address these challenges, the researchers prepared superhydrophobic coatings with excellent acid and alkali resistance, ice resistance and self-cleaning capability by adding nanoparticles such as SiO_2_ [28,29], TiO_2_ [30,31], ZnO [32,33], Fe_3_O_4_ [34,35] and Al_2_O_3_ [36,37] to various polymer matrices. Yang et al. [38] synthesized a superhydrophobic coating by combining ZrO_2_ with polydimethylsiloxane (PDMS). This PDMS-ZrO_2_ composite coating exhibited outstanding corrosion resistance. Celik et al. [39] developed a superhydrophobic coating with remarkable corrosion resistance and self-cleaning properties. They achieved this by the ball milling process inducing a reaction between dodecyltrichlor-osilane’s trichloroalkysilane and hydrophilic silica nanoparticles, leading to chemical grafting. Superhydrophobic coatings have been successfully utilized in anti-icing applications. Specifically, Qi et al. [40] employed an etching technique to evenly distribute SAN particles, dissolved by ASA resin, within a THF and EtOH solvent mixture to fabricate a hydrophobic surface. Notably, at a chilling ambient temperature of −10 degrees Celsius, the time taken for water droplets to crystallize on this coated surface was significantly prolonged to 63 min, indicating superior anti-icing efficacy.

To prepare wear-resistant superhydrophobic coatings using fillers in resin, two main methods are employed. The first approach involves directly incorporating fillers into the resin, thereby creating a coarse surface texture. Here, the filler becomes entirely wetted by the resin. This results in the development of a surface roughness predominantly via particles, while the resin contributes to the reduced surface energy. This synergy enables super hydrophobicity. A key advantage of this technique is that the introduction of ample fillers ensures a uniform rough structure both internally and externally. Coupled with the intrinsic hydrophobic nature of the resin, this ensures that the coating retains its superhydrophobic property until completely worn out. This method is presently the dominant means of achieving super hydrophobicity using fillers. However, its downside is that the film’s overall strength diminishes due to the extensive filler content, leading to hydrophobicity degradation when sanded. Furthermore, it exhibits poor resistance to impacts and abrasions, causing the coating to detach easily from the substrate.

The alternative approach is to use the “adhesive + particle” [41,42] method, utilizing potent adhesives like epoxy resin and water-based polyurethane to bind hard hydrophobic particles directly onto the coating, providing friction resistance in a robust manner. In this scenario, the particles serve dual functions: bestowing reduced surface energy and imparting surface roughness. The superhydrophobic coatings derived via this method exhibit enhanced hydrophobicity and, given the minimal particle incorporation within adhesive, have superior post-film formation strength.

Interestingly, there seems to be an oversight in current research regarding the potential of ZrO_2_ nanoparticles. Furthermore, many current methods demand intricate steps, high-tech equipment, and can be operationally challenging, especially when contrasted with the industry-preferred spray coating technique. To bridge this gap, our research introduces a novel coating formulation. Some silicone-modified acrylic emulsion was pre-introduced into the ethanol suspension. By incorporating SiO_2_ particles and ZrO_2_ nanoparticles modified with the silane coupling agent KH-570, into the ethanol mixture, we achieved diverse composite coatings. With the strategic use of silicone-modified acrylic emulsion and selected nanoparticles, we developed a superhydrophobic coating via the spray coating technique. The rigorous assessment addressed wear resistance, adaptability in acidic and alkaline conditions, durability against sodium chloride exposure, self-cleaning properties, and anti-icing effectiveness. The results were promising, showcasing the coatings’ potential for real-world implementations.

## 2. Materials and Methods

### 2.1. Material

SiO_2_ particles, with diameters ranging from 1 to 10 μm, were procured from Yichang Huifu (Yichang, China). ZrO_2_ nanoparticles, characterized by a diameter range of 20–50 nm, came from Macklin Reagents (Shanghai, China). The silane coupling agent KH-570 (γ-Methacryloxypropyltrimethoxysilane) was utilized from Macklin Reagents (Shanghai, China). The silicone-modified acrylic emulsion was produced by Anhui Tengtuo (Chizhou, China). AR-grade toluene was provided by Tianjin Fuyu Fine Chemicals (Tianjin, China). Both ethanol and hydrochloric acid (36~38%) of AR-grade were secured from Guoyao (Shanghai, China). Sodium hydroxide of AR grade was sourced from Macklin Reagents (Shanghai, China), while AR-grade sodium chloride was supplied by Xilong (Shantou, China). All experiments used self-prepared ultrapure deionized water.

### 2.2. Modification of ZrO_2_ Nanoparticles

Modified ZrO_2_ nanoparticles were hydrophobically enhanced using silane coupling agent KH-570. The modification process began with the ZrO_2_ nanoparticles being laid out in a sterile Petri dish. These particles were then dried at 60 °C in an electric drum air oven for 6 h. Subsequently, 4 g of these ZrO_2_ nanoparticles were combined with 30 mL of toluene. This mixture was stirred at room temperature for 20 min, followed by 20 min of ultrasonic agitation. Afterward, 20 mL of a 10% concentration of silane coupling agent KH-570 was added, and the entire blend underwent 10 min of ultrasonic dispersion. This dispersion was transferred to a 250 mL three-necked flask and was agitated at 80 °C for 30 min. Post cooling to room temperature, the mixture underwent centrifugation at 10,000 r·min^−1^ for 30 min. The resulting substance was filtered using a vacuum filtration method to obtain the modified ZrO_2_ nanoparticles. These were then dried in a vacuum oven at 100 °C for 6 h.

### 2.3. Preparation of ZrO_2_/SiO_2_/Silicone-Modified Acrylic Emulsion Superhydrophobic Coating

Adopting a spray technique to fabricate superhydrophobic coatings. Initially, 0.1 mL of silicone-modified acrylic emulsion was dissolved in 1 mL of ethanol. Following this, 0.04 g of modified ZrO_2_ nanoparticles and 0.06 g of SiO_2_ particles were added to the solution. The mixture was magnetically stirred for 20 min at ambient temperature and then ultrasonically dispersed for an additional 5 min, producing ethanol dispersion with inorganic particles.

**Figure 1:**
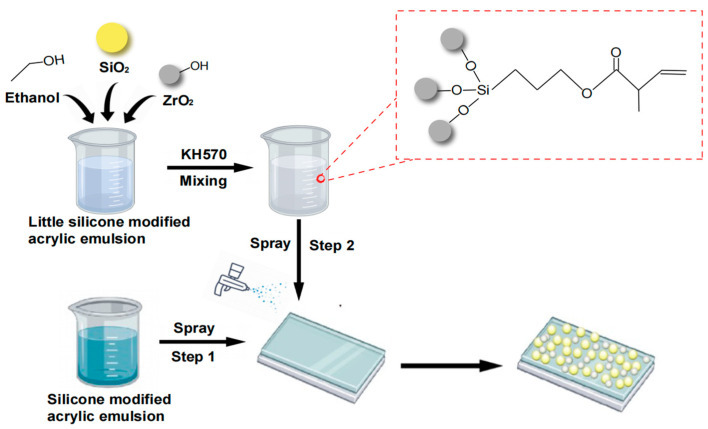
Schematic diagram of the preparation process of coating.

In the next phase, a superhydrophobic coating was created. This was achieved by spraying 2 mL of the silicone-modified acrylic emulsion evenly at 4 MPa onto a 2.5 × 7.5 cm slide and then spraying zirconia silica ethanol dispersion. Subsequently, the coated sample was cured at 100 °C for 20 min. The unattached particles on the coating’s surface were removed by air flow and the obtained coating was named MZS-0.1. By using a similar procedure, MZS-0 and MZS-0.2 were prepared by using 0 and 0.2 mL silicone-modified acrylic emulsion, respectively. For comparison, MS-0.1 was also prepared without modified ZrO_2_ nanoparticles. The entire process is illustrated in Figure 1. The surface of ZrO_2_ inorganic particles features a Zr-O-H structure, which interacts with the Si-O-H structure of KH-570, leading to the formation of a modified ZrO_2_ with a Zr-O-Si structure. Additionally, KH-570 possesses a carbon-carbon double bond. This double bond undergoes a reaction with the corresponding double bond in the silicone-modified acrylic emulsion, resulting in the creation of a novel covalent bond.

Due to the water-based nature of the emulsion, it vigorously repels hydrophobic particles, making wetting challenging. Additionally, the emulsion’s adhesion capacity is inferior in comparison to potent adhesives like epoxy resin. In conclusion, the emulsion’s modest hydrophobic nature makes it challenging to achieve super hydrophobicity using the first method (directly incorporating fillers into resin). Direct coatings prepared by the second method (adhesive + particle) demonstrated subpar wear resistance. During experimentation, a uniform silicone-modified acrylic emulsion (2 mL) was initially sprayed onto the glass substrate, followed by applying an ethanol suspension of particles application, with particles constituting about 5% of the coating’s mass fraction. Some silicone-modified acrylic emulsion was pre-introduced into the ethanol suspension, enhancing the inter-particle bonding strength. Ethanol’s rapid evaporation during curing ensures the resin and particles intertwine. Adding a specific resin quantity to the particle suspension before curing accelerates this intermingling, enabling a cure at 100 °C for 20 min, forming a dispersed transition zone between the particles and the resin. The emergence of this zone ensures hydrophilic nanoparticles and hydrophobic silicone-modified acrylic emulsion interpenetrate and adhere securely to the glass substrate.

### 2.4. Chemical Structure Characterization

In this study, Nexus 670 FTIR instrument (Thermo Nicolet, MA, USA) was used to analyze the functional groups of ZrO_2_ nanoparticles both before and after modification, and the KBr pressing technique was employed. The crystalline structures of the ZrO_2_ nanoparticles, before and after modification, were assessed through X-ray diffraction (XRD) diagrams captured on the SmartLab^TM^ X-ray diffractometer (Rigaku, Tokyo, Japan) set at 3 kW. Energy dispersive spectroscopy (EDS) was investigated using a JSM-6510 scanning electron microscopy (JEOL, Tokyo, Japan), which elucidated the elemental composition of the modified ZrO_2_ nanoparticle surface. To probe the micro-nanometric structure of the ZrO_2_/SiO_2_/silane–acrylic acid modified emulsion superhydrophobic coating, coat a layer of gold on the surface of the characterization sample and dry it in a vacuum. After the sample was prepared, SEM images were taken using a JSM-6510 scanning electron microscope (JEOL, Tokyo, Japan) with a 20 kV acceleration voltage. Additionally, the OLA4000 laser scanning confocal microscope (Olympus, Tokyo, Japan) provided three-dimensional visualizations of the diverse compositions present on the surface of the ZrO_2_/SiO_2_/silane–acrylic acid modified emulsion superhydrophobic coating.

### 2.5. Measure the Contact and Rolling Angles

Contact angles and rolling angles were analyzed by using an SDC-350 contact angle measuring instrument (Dongguan Shengding Precision Instrument, Dongguan, China). the contact angles for all samples were measured at room temperature involved taking an average of five measurements with 6 µL water droplets dropping from 50 mm height. In the case of composite coatings with different compositions, the contact angle was measured directly on the surface using the same procedure. Additionally, the rolling angle for all composite coatings was determined using the same contact angle instrument. For this measurement, samples were stabilized on the instrument platform. The rolling angle of 6 µL water droplets was ascertained by tilting the platform and altering its angle relative to the horizontal plane, until the droplets commenced sliding.

### 2.6. Wear Resistance

During the sandpaper abrasion test, a glass substrate coated with the ZrO_2_/SiO_2_/silane–acrylic acid modified emulsion superhydrophobic layer underwent polishing using a 2000 # sandpaper. For each testing cycle, samples are pressed by a 200 g weight and slide 10 cm horizontally along the ruler. After a series of equidistant abrasion cycles, the contact and rolling angles of the coatings with different compositions were evaluated.

### 2.7. Chemical Stability

Researchers conducted two distinct immersion tests on superhydrophobic coatings with varying compositions. In the acid resistance test, we completely immersed nine samples of identical composition in hydrochloric acid (pH = 1, 0.1 mol/L). Every hour, we assessed the contact angle and rolling angle of one sample. Similarly, in the alkali resistance test, nine samples were fully immersed in a sodium hydroxide solution (pH = 14, 1 mol/L), with the contact angle and rolling angle of one sample evaluated hourly. For the salt tolerance test, seven samples were immersed in a 3.5% sodium chloride solution, and we measured the contact angle and rolling angle of one sample every 6 h.

### 2.8. Self-Cleaning Performance

Identical thicknesses of graphite powder and sand particles were sprinkled onto the surfaces of superhydrophobic coatings with varied compositions. To evaluate the self-cleaning capability, the coatings were tilted at a tilt angle of approximate 30°, and the ability of equal volume water droplets to roll off, collecting the contaminants, was observed. Additionally, the potential of these coatings to prevent stains was assessed by observing if a consistent volume of coffee solution left any residues when passed over them.

### 2.9. Anti-Icing Performance

50 μL water droplets were applied to a glass substrate coated with superhydrophobic materials of various compositions. This setup was placed inside a BL-LS400CDB refrigerator (Yeqi Electric Appliance, Shanghai, China), With a consistent cooling rate of 1 °C/min, cooling commenced from 0 °C. Throughout the process, a camera (image capture rate: 30 fps) was employed to capture the transition of water droplets to ice.

### 2.10. Water Droplet Bouncing, Water Flow Bending and Underwater Silver Light Reflection Performance

A water droplet of 6 μL volume and the water jet were observed to investigate the movement of it when touching the coating surface. Additionally, a unique silver reflection phenomenon associated with the superhydrophobic coating was noted. All fundamental images were captured by using an SDC-350 contact angle measuring instrument.

## 3. Results

### 3.1. Characterization of Modified ZrO_2_ Nanoparticles

#### 3.1.1. Microstructure

**Figure 2:**
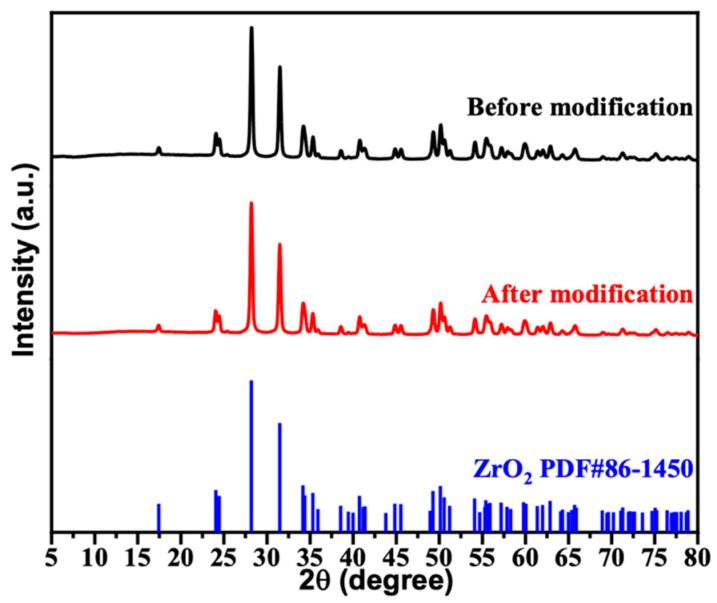
XRD diffraction pattern of zirconium dioxide.

As shown in Figure 2, the pre-modified and modified ZrO_2_ to XRD tests, and the observed peaks of the XRD pattern are indexed and compared with pure ZrO_2_ (PDF#86-1450). It can be seen that the profile contains distinct characteristic peaks at 2𝚹 angles (24°, 28°, 31°, 34°, and 50°), which represent the optimal orientations of the five crystallographic planes (1 1 0), (−1 1 1), (1 1 1), (0 0 2), and (2 2 0), respectively. This XRD spectrum not only confirms the monoclinic crystal system of nano-ZrO_2_, but also indicates its own high crystallinity by its narrower linewidth [43]. More notably, the diffraction peaks before and after the modification remain consistent, indicating that the structural integrity of the ZrO_2_ nanoparticles is largely unaffected by the surface modification.

**Figure 3:**
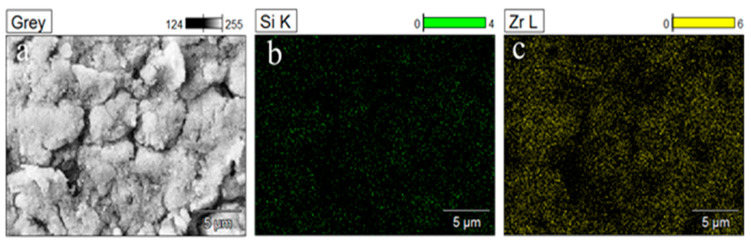
(**a**) SEM secondary electron image of modified zirconium dioxide, magnified 3000 times; (**b**) distribution of Si element in modified ZrO_2_ nanoparticles; (**c**) distribution of Zr element in modified ZrO_2_ nanoparticles.

Figure 3 presents the SEM image of the modified ZrO_2_, where the even dispersion of the Si element from the modifier KH-570 is evident. This uniform distribution confirms the successful modification of ZrO_2_. Furthermore, the coating surface displays a distinct rough and porous texture.

#### 3.1.2. Chemical Composition

**Figure 4:**
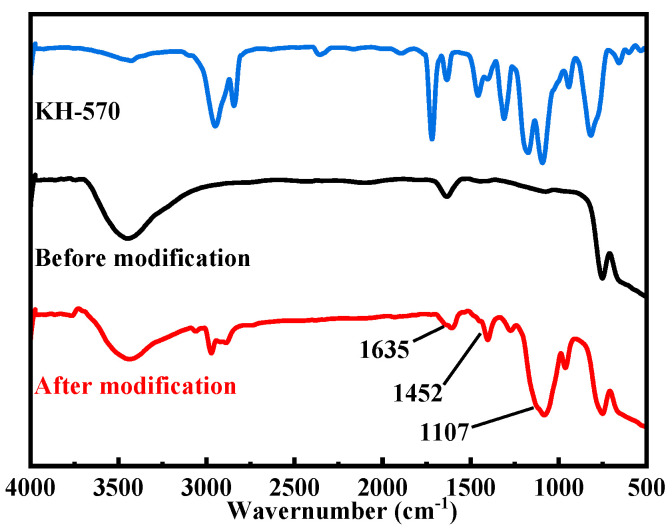
Infrared spectrum of ZrO_2_ and KH-570.

Figure 4 presents the FTIR spectrum of ZrO_2_ nanoparticles pre and post-modification. The modified ZrO_2_ spectrum exhibits distinctive peaks: the one at 1107 cm^−1^ arises from the asymmetric stretching vibration of the -Si-O-C- group, the 1452 cm^−1^ peak represents the stretching vibration of the -COO- group, and the peak at 1635 cm^−1^ is associated with the C=C double bond stretching vibration. These peaks validate the dehydration reaction between hydrolyzed methacryloyloxypropyltrimethoxysilane molecules and ZrO_2_ nanoparticles, corroborating the modification’s success. This altered the original hydroxyl-rich, high-porosity coating to a surface with reduced energy, preventing pollutant infiltration and bolstering the coating’s environmental stability [44].

### 3.2. Characterization of ZrO_2_/SiO_2_/Silicone-Modified Acrylic Emulsion Superhydrophobic Coatings with Different Components

#### 3.2.1. Micromorphology

**Figure 5:**
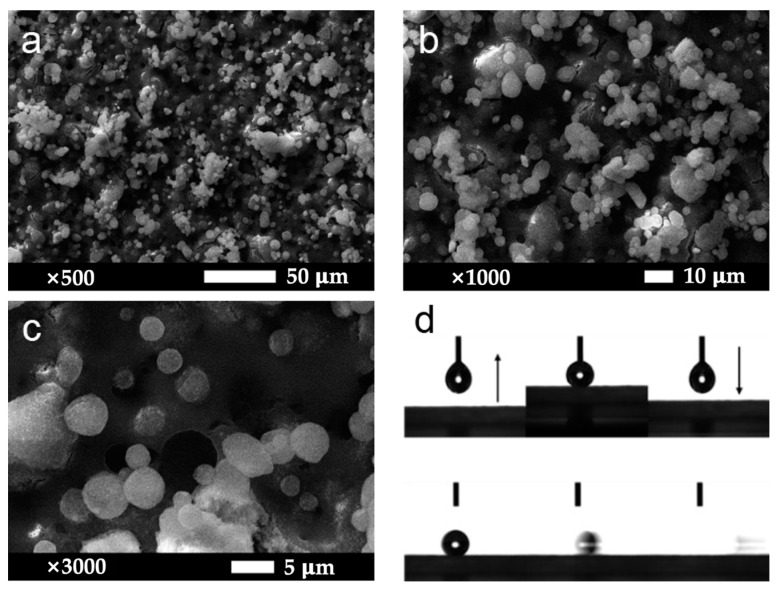
(**a**–**c**) Microscopic morphology images of the MZS-0.1 coating surface, magnified 500 times, 1000 times, and 3000 times in order; (**d**) the 8 μL droplet cannot be directly picked up with the MZS-0.1 superhydrophobic coating, and the droplet is very easy to roll on the MZS-0.1 coating.

**Figure 6:**
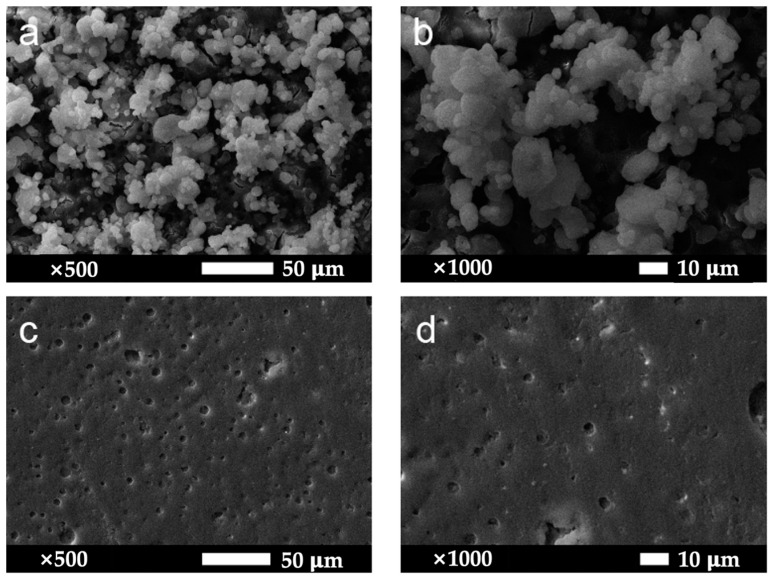
(**a**,**b**) Microscopic morphology images of the MS-0.1 coating surface, magnified 500 times, 1000 times in order; (**c**,**d**) images of the surface micromorphology of pure silicone-modified acrylic emulsion coating, magnified 500 times and 1000 times, respectively.

Figure 5 and Figure 6 display SEM images of three different coatings. Microscopic morphology of the MZS-0.1 coating surface is shown in Figure 5a–c. The modified ZrO_2_ nanoparticles cluster on the surface, creating large, distinctly aggregate spheres with substantial gaps between them. This micro-nano structure efficiently captures air while repelling water molecules, ensuring the coating’s superhydrophobic nature. Figure 5d demonstrates that droplets cannot directly associate with the MZS-0.1 superhydrophobic coating and easily roll off its surface.

Figure 6a,b illustrates the surface morphology of the MS-0.1 coating, revealing that it is less effective than the MZS-0.1 coating in creating air layer voids critical for super-hydrophobicity. Larger particle sizes in MS-0.1 coating result in less surface voids, which adversely affect the nanostructure essential for optimal performance. To preserve the essential micro-nano structure in the superhydrophobic coating, it is necessary to mix modified ZrO_2_ nanoparticles with SiO_2_. This blend may improve the distribution of initially clustered particles and encourage the formation of large clusters and voids. In contrast, as observed in Figure 6c,d, the pure silane-modified emulsion coating displays a smoother surface devoid of the aforementioned micro-nano structures.

**Figure 7:**
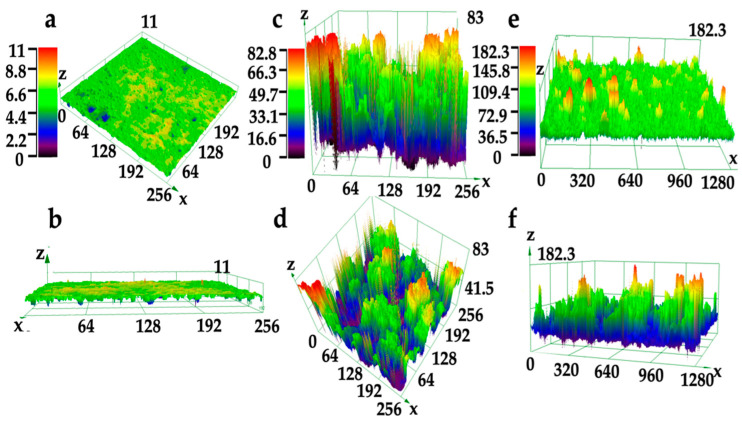
The CLSM images of various coatings: (**a**,**b**) pure silicone-modified acrylic emulsion coating surface; (**c**,**d**) MS-0.1 coating surface; (**e**,**f**) MZS-0.1 coating surface.

Figure 7 presents the CLSM images of various coatings, aligning with the SEM findings. The MZS-0.1 coating’s surface (Figure 7e,f) displays pronounced orderly convex frame, especially when juxtaposed with the smoother surface of the pure silane-modified emulsion coating (Figure 7a,b). Compared with pure silicone-modified acrylic emulsion coating (height differential of less than 10 μm), the MZS-0.1 coating showed a height disparity reaching nearly 100 μm). Additionally, the MS-0.1 coating showed a height disparity reaching approximate 70 μm. This may be due to micro/nano hierarchical structure of SiO_2_/ZrO_2_. The surface of the MS-0.1 coating, as illustrated in Figure 7c,d, exhibits a less height differential than MZS-0.1 coating, which is not conducive to the formation of suitable sized voids for capturing air.

#### 3.2.2. Hydrophobicity

**Figure 8:**
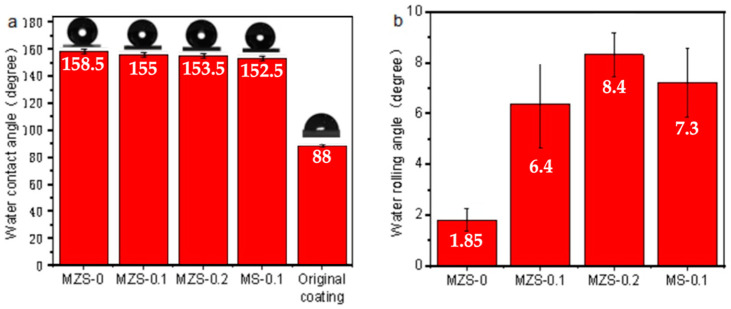
(**a**) Contact angle of different coatings; (**b**) rolling angle of different coatings.

Figure 8 presents the contact and rolling angles for four coatings: MZS-0, MZS-0.1, MZS-0.2, and MS-0.1. The data indicate that MZS-0 exhibits superhydrophobic properties, whereas the remaining coatings—MZS-0.1, MZS-0.2, and MS-0.1—display only partial hydrophobicity, as evidenced by their rolling angles ranging from 5 to 10 degrees.

As the silicone-modified acrylic emulsion content increases, the superhydrophobic coating’s hydrophobic nature decreases, evident from the increasing roll-off angle. The contact angle of the pure silane-modified emulsion coating registers at 88° relative to the horizontal plane, lacking any superhydrophobic traits. It is evident that the silane-modified emulsion’s over-wetting of hydrophobic particles results in a diminished contact angle and a heightened rolling angle. This phenomenon is attributed to the high surface free energy of the silicone-modified acrylic emulsion, which increases the superhydrophobic coating’s high surface free energy. Concurrently, excessive silicone-modified acrylic emulsion infiltration into surface ZrO_2_/SiO_2_ nanoparticles causes underexposure of these nanoparticles, compromising the micro-nano surface structure and subsequently decreasing the superhydrophobic coating’s surface roughness [45].

The comparison of coating contact angle and rolling angle is shown in Table 1 [46,47,48,49,50] where ZrO_2_/SiO_2_/silicone-modified acrylic emulsion coating has a significant hydrophobic effect.

### 3.3. Wear Resistance of ZrO_2_/SiO_2_/Silicone–Acrylic Acid Modified Emulsion Superhydrophobic Coating

**Figure 9:**
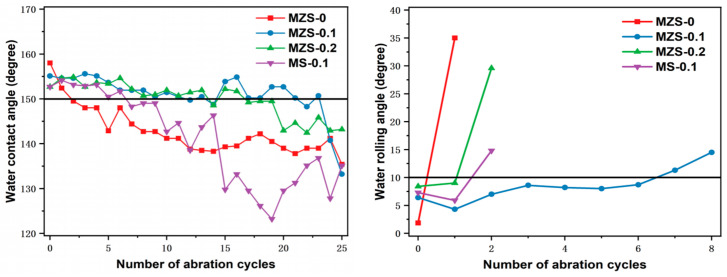
Wear resistance test of different organic hydrophobic coatings.

Figure 9 presents the contact and rolling angles of coatings with varying compositions after enduring consistent wear and tear. Given the same wear frequency, the MZS-0.1 coating’s hydrophobicity declines after six wear cycles. In contrast, the MZS-0 coating with pure ZrO_2_/SiO_2_, without the addition of silicone-modified acrylic emulsion, maintains its rolling angle only once. After three successive wears, its contact angle drops below 150°, indicating a swift loss of super-hydrophobicity. This highlights the crucial role of silicone-modified acrylic emulsion in bolstering the bond between the substrate and ZrO_2_/SiO_2_ surface nanoparticles, as well as the cohesion amongst the nanoparticles themselves. The contact angle trajectory for each coating remains predominantly stable, which can be ascribed to the transition region between the nanoparticle and silicone-modified acrylic emulsion layers arising from ethanol’s evaporation. Within this zone, the sandpaper-induced texture post-rubbing closely mirrors its initial state, thus preserving super-hydrophobicity. Hence, integrating silicone-modified acrylic emulsion with nanoparticle ethanol dispersions can somewhat mitigate friction-induced hydrophobicity reduction. Yet, an excessive infusion of silicone-modified acrylic emulsion can escalate the particle dispersion’s viscosity, complicating the spraying process and potentially leading to issues like cracking.

Upon incorporating the silicone-modified acrylic emulsion, the MS-0.1 coating loses its super-hydrophobicity post roughly one wear. However, the coatings embedded with ZrO_2_ nanoparticles exhibited good wear resistance; notably, the MZS-0.1 coating maintained its super-hydrophobicity after six wear cycles. The superior hardness of ZrO_2_ nanoparticles, compared to SiO_2_ particles, suggests that ZrO_2_ inclusion amplifies the superhydrophobic coating’s resistance to wear.

### 3.4. Corrosion Resistance of ZrO_2_/SiO_2_/Silicone-Modified Acrylic Emulsion Superhydrophobic Coating

#### 3.4.1. Acid and Alkali Corrosion Resistance

**Figure 10:**
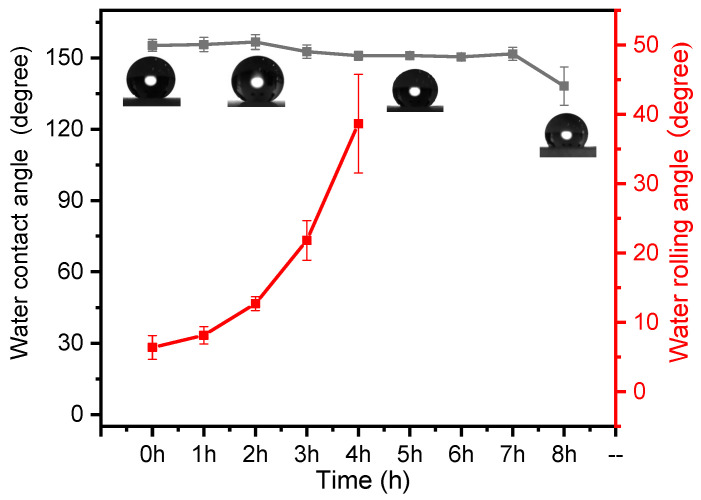
Changes in contact angle and rolling angle of MZS-0.1 coating in dilute hydrochloric acid solution with pH = 1.

Figure 10 illustrates the changes in contact and rolling angles of the MZS-0.1 coating under exposure to a highly acidic environment with a pH value of 1. Notably, the MZS-0.1 coating’s hydrophobicity declines within 1–2 h, as indicated by the increase in the rolling angle to above 5 degrees during this period. In comparison to the contact angle, the rolling angle is more susceptible to shifts in the surface’s roughness and chemical composition of low-energy materials. Even trivial wear and corrosion can lead to pronounced discrepancies in the rolling angle. This heightened corrosion resistance in superhydrophobic coatings is attributed to the presence of a surface air layer, functioning as a shield against corrosive substances. This prevents these substances from infiltrating the coating, interacting with the inorganic nanoparticles, and damaging the surface’s micro-nano structures. With the time passing, the superhydrophobic coating loses its unique properties.

**Figure 11:**
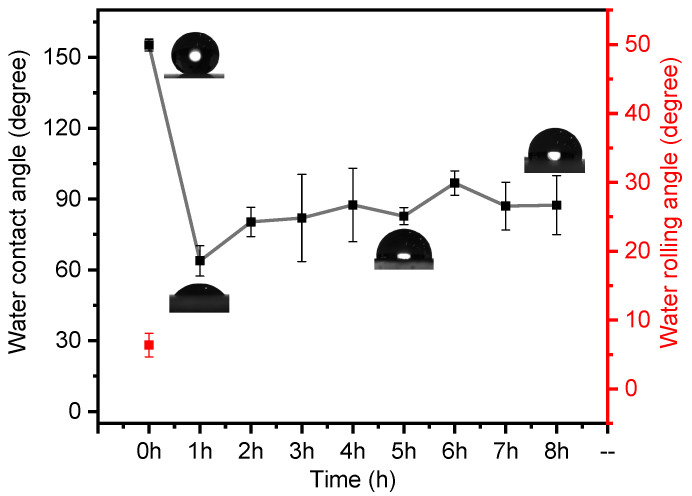
MZS-0.1 coating in sodium hydroxide solution with pH = 14.

On the other hand, as depicted in Figure 11, the MZS-0.1 coating does not demonstrate significant resistance to alkalinity in a potent alkaline environment with a pH of 14. The hydrophobic characteristics of the MZS-0.1 coating are entirely compromised within just an hour. The contact angle of the destroyed MZS-0.1 coating (63°) strays considerably from the 88° typical of pure silicone-modified acrylic emulsion coatings. This can be attributed to the potent alkali disrupting the chemical bonds between the surface’s SiO_2_ and ZrO_2_ nanoparticles, which are interconnected by low surface energy organic polymers. This interference causes the initially hydrophobic particles to revert to a hydrophilic state, turning the hydrophobic coating more hydrophilic. Consequently, the water contact angle for these hydrophilic particles is notably lower than that of the pure silicone-modified acrylic emulsion. After a 3 h immersion, the hydrophobic coating begins separating from its glass substrate. These observations suggest that alkali can destroy the interactions between hydrophobic coating and glass substrate.

#### 3.4.2. Sodium Chloride Corrosion Resistance

**Figure 12:**
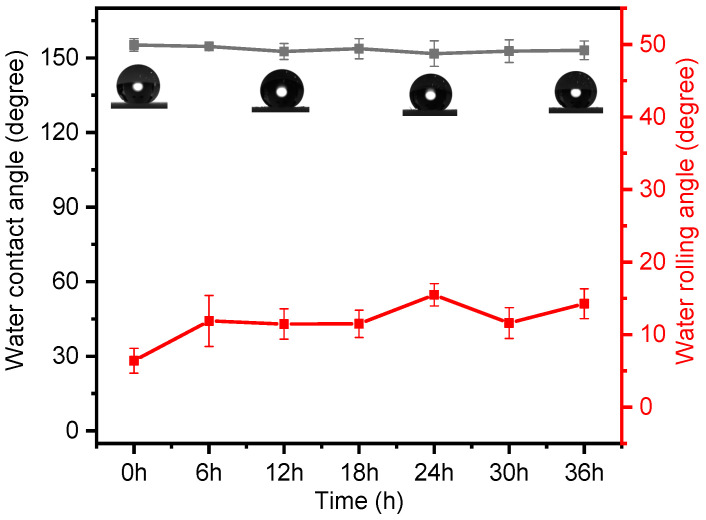
MZS-0.1 coating in 3.5% mass fraction sodium chloride solution.

Figure 12 presents the contact and rolling angles of the MZS-0.1 coating after varying durations of immersion in sodium chloride. The data illustrate that with an increase in immersion time, the MZS-0.1 coating’s contact angle remains relatively stable at approximately 152°. However, it is observed that the rolling angle of the MZS-0.1 coating displayed significant variance with prolonged immersion, and its hydrophobicity descends after 6 h. This suggests minimal corrosion impact on the MZS-0.1 coating from the sodium chloride solution. In summary, the MZS-0.1 coating undeniably demonstrates superior resistance against sodium chloride corrosion.

### 3.5. Self-Cleaning Properties of ZrO_2_/SiO_2_/Silicone-Modified Acrylic Emulsion Superhydrophobic Coating

**Figure 13:**
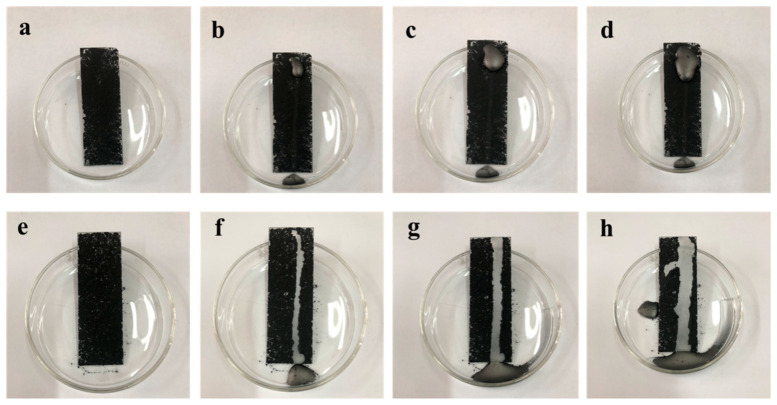
(**a**–**d**) Images of cleaning graphite powder on the surface of pure silicone–acrylic-modified emulsion coating; (**e**–**h**) images of graphite powder cleaning on MZS-0.1 coating surface.

Figure 13 illustrates a images representation of graphite powder blanketing the surface of both the pure silicone-modified acrylic emulsion coating and the MZS-0.1 coating at a tilt angle of approximate 30°. Upon dropping the same quantity of distilled water droplets, it is evident that the water beads clump together and find it challenging to roll off the pure silicone-modified acrylic emulsion coating. This behavior indicates a lack of self-cleaning attributes in the pure silicone-modified acrylic emulsion. Conversely, water droplets roll effortlessly on the MZS-0.1 coating, effectively shedding any attached dirt, highlighting the MZS-0.1 coating’s superior self-cleaning capabilities.

**Figure 14:**
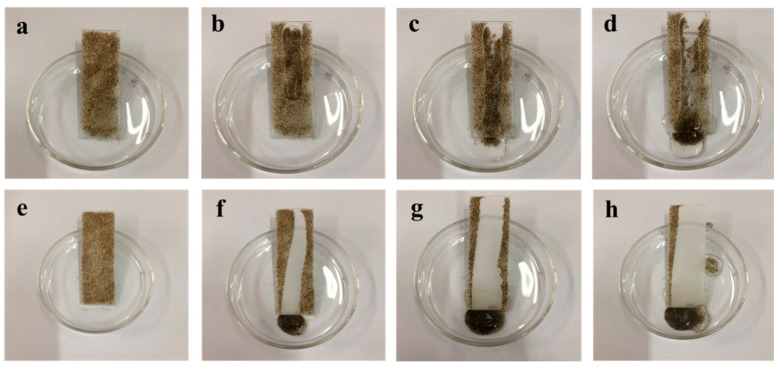
(**a**–**d**) Images of cleaning sand particles on the surface of pure silicone-modified acrylic emulsion coating; (**e**–**h**) images of cleaning sand particles on the surface of MZS-0.1 coating.

Figure 14 depicts the cleaning process at a tilt angle of approximate 30° with identical distilled water droplet quantities on both the pure silicone-modified acrylic emulsion and MZS-0.1 coatings, both coated evenly with sand. The MZS-0.1 coating demonstrates a substantially more effective cleaning capacity than its pure silicone-modified acrylic emulsion counterpart, reiterating its excellent self-cleaning features.

**Figure 15:**
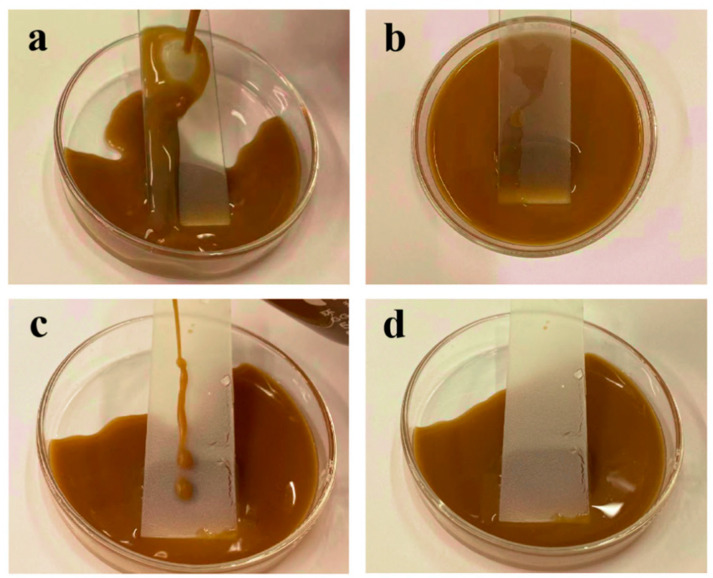
(**a**,**b**) Images of surface contamination of pure silicone-modified acrylic emulsion coating; (**c**,**d**) images of surface contamination of MZS-0.1 coating.

In another test, shown in Figure 15, the same amount of sticky coffee liquid is poured at a tilt angle of approximate 30° onto both coatings. The MZS-0.1 coating impressively ensures that the coffee droplets glided off without leaving any residue, maintaining its uncontaminated state. On the other hand, evident marks are left behind by the coffee droplets on the pure silicone-modified acrylic emulsion coating, signifying some level of contamination.

In light of the above observations, the coating has a good self-cleaning effect on solid particles such as graphite powder and fine sand particles and liquid pollutants such as coffee.

### 3.6. Anti-Icing Performance of ZrO_2_/SiO_2_/Silicone-Modified Acrylic Emulsion Superhydrophobic Coating

**Figure 16:**
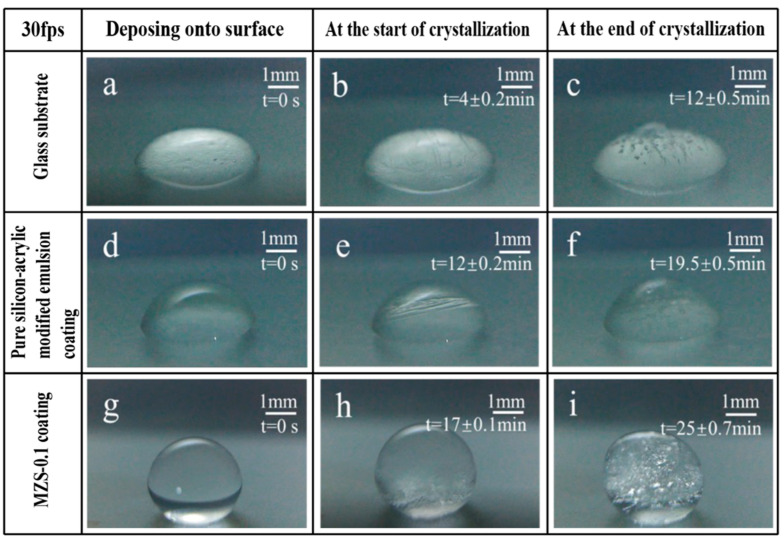
(**a**–**c**) Freezing process of a water droplet on glass substrate surfaces; (**d**–**f**) freezing process of a water droplet on pure silicon-acrylic modified emulsion coating surfaces; (**g**–**i**) freezing process of a water droplet on MZS-0.1 coating surfaces.

Following the anti-icing evaluations on glass substrates, pure silicone-modified acrylic emulsion coatings, and MZS-0.1 coatings (as depicted in Figure 16), the initial time of ice formation serves as a pivotal metric in assessing the coatings’ anti-icing capabilities. When cooled at a steady rate of 1 °C/min starting from 0 °C, the crystallization time of water droplets on a glass substrate is approximately 4 min. In contrast, this time extends to 12 min for the pure silicone-modified emulsion coating—threefold longer than that of the glass—and to 17 min for the MZS-0.1 coating, which is over four times the duration of the glass substrate. All three samples reach a completely frozen state roughly eight minutes following the appearance of ice crystals.

The smaller contact area between the spherical droplets and the coated surface leads to a reduced heat transfer rate and the presence of an air layer, which diminishes the direct contact between the water droplets and the superhydrophobic coating. This effectively decreases the likelihood of heterogeneous nucleation. Furthermore, crystallization kinetics principles suggest that the droplets on the superhydrophobic surface must overcome a significantly larger Gibbs free energy barrier to crystallize, particularly through heterogeneous nucleation. The magnitude of this barrier correlates with the increase in contact angle [51]. Consequently, the presence of modified ZrO_2_ on the coating’s surface substantially delays the crystallization time, suggesting that the coating possesses valuable anti-icing properties.

### 3.7. Water Droplet Bouncing, Water Flow Bending and Underwater Silver Light Reflection Properties of ZrO_2_/SiO_2_/Silicone-Modified Acrylic Emulsion Superhydrophobic Coating

**Figure 17:**
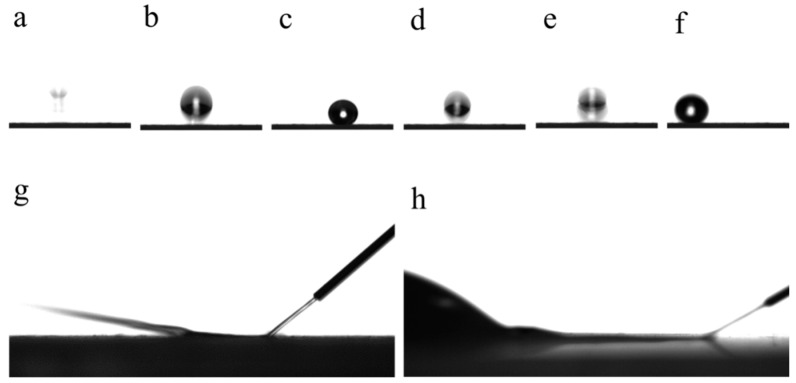
(**a**–**f**) Images of a 6 μL water droplet bouncing vertically on the MZS-0.1 coating from a height of 20 mm; (**g**) photo of water flow bending on the surface of MZS-0.1 coating; (**h**) photo of water flow accumulating on the surface of pure silicone-modified acrylic emulsion coating.

A 6 μL water droplet exhibits pronounced elasticity when placed on the MZS-0.1 coating, as illustrated in Figure 17a–f, demonstrating a distinct bouncing effect [52]. When water is introduced to this coating using a syringe, it ricochets, similar to a laser beam reflecting off a mirror, as seen in Figure 17g. The air layer, residing on the surface, contributes to ricocheted behavior and enhances the elasticity observed in water droplets.

**Figure 18:**
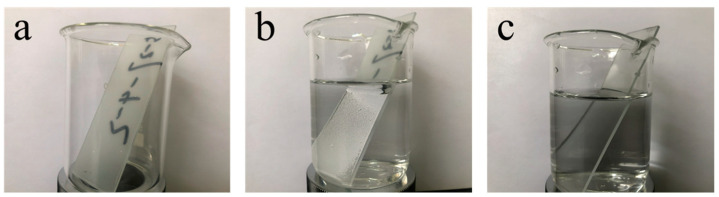
Photos of silver light reflection of superhydrophobic coating underwater; (**a**) MZS-0.1 coating in air; (**b**) MZS-0.1 coating in water; (**c**) pure silicone-modified acrylic emulsion coating in water.

Figure 18 captures the underwater luminous reflection exhibited by the MZS-0.1 coating. When the superhydrophobic coating is submerged, the surface’s air layer produces a lustrous, silver-like light, attributed to interface reflection. This interface reflection is intrinsically linked to the air layer on the surface.

In conclusion, the surface air layer is pivotal in enhancing the self-cleaning, corrosion resistance, drag minimization, and anti-icing attributes of superhydrophobic coatings. It supports several of the notable properties of these coatings [53]. A compromised surface air layer typically indicates that the superhydrophobic coating has been fully saturated, leading to the forfeiture of its superhydrophobic attributes [54].

## 4. Conclusions

In summary, we have successfully developed a method to fabricate four distinct coatings (MZS-0, MZS-0.1, MZS-0.2, MS-0.1) on glass substrates. This was achieved using a unique blend of γ-Methacryloyloxypropyltrimethoxysilane (KH570) modified ZrO_2_/SiO_2_/silicone-modified acrylic emulsion to produce a superhydrophobic emulsion coating. One coating achieved super-hydrophobicity, registering a contact angle of 158.5 degrees and a rolling angle of 1.85 degrees. The inherent bonding strength of the silicone-modified acrylic emulsion ensures that these coatings can endure extensive mechanical wear, evident from their resistance to more than six abrasion cycles using 2000-grit sandpaper. Remarkably, the MZS-0.1 variant retains its superhydrophobic properties under extreme conditions, such as a 2 h immersion in strong acid (pH = 1, 0.1 mol/L) or a 6 h soak in salt water. Moreover, the MZS-0.1 coating showcases superior self-cleaning capabilities in an aerial environment. Due to the inclusion of KH570, the modified ZrO_2_ surface significantly delays the crystallization of water droplets to 17 min, indicating a measure of anti-icing capability. This coating is also characterized by its surface elasticity and a distinctive underwater silvery sheen. The investigation into these coatings may reveal innovative approaches for developing effective superhydrophobic surfaces, specifically through the strategic use of emulsion mixtures with ZrO_2_ and SiO_2_.

## Figures and Tables

**Table 1:** Comparisons of different coatings by contact angle and rolling angle tests.

Coating Composition	Preparation Process	Contact Angle (CA)	Rolling Angle (RA)	Ref.
316L/nano-TiO_2_/TMPSi	one-step EPD	168°	3.1°	[46]
PS/OTS/SS	Sol-gel	157.5°	6°	[47]
MTES/SiO_2_/MOH	Sol-gel	153°	9°	[48]
PDMS/MTCS/SiO_2_/TiO_2_	Spray	151°	9°	[49]
F-PE/SiO_2_	Template	158°	4°	[50]
ZrO_2_/SiO_2_/siloxane-modified acrylic emulsion	Spray	158.5°	1.85°	This work

## Data Availability

Data are contained within the article.
